# The epistemological role of empathy in psychopathological diagnosis: a contemporary reassessment of Karl Jaspers’ account

**DOI:** 10.1186/1747-5341-9-6

**Published:** 2014-03-17

**Authors:** Panagiotis Oulis

**Affiliations:** 1 First Department of Psychiatry, University of Athens, Eginition Hospital, 72-74 Vas. Sophias Avenue, Athens 11528, Greece

**Keywords:** Karl Jaspers, Phenomenological approach in psychopathology, Method of empathy, Clinical epistemology, Philosophy of science, Philosophy of psychiatry

## Abstract

**Introduction:**

In his classic essay “The phenomenological approach to psychopathology”, Karl Jaspers defended the irreducible reality of the “subjective” mental symptoms and stressed the pivotal role of empathy in their diagnostic assessment. However, Jaspers’ account of the epistemological role of empathy in psychopathological diagnosis was far from clear: whereas at several places Jaspers claimed that empathy provides a direct access to patients’ abnormal mental experiences, at other places he stressed that it did so only indirectly, through a whole battery of their observable clinical indicators. The aim of this paper is to reassess Jaspers’ account of the epistemological role of empathy in psychopathological diagnosis.

**Methods:**

I examine thoroughly Jaspers’ assertions on in the role of empathy in the diagnosis of “subjective” symptoms. Moreover, I explicate briefly the epistemological status of psychopathological diagnostic examination with the aid of the distinction between direct and indirect observation.

**Results:**

Diagnostic assessment of “subjective” mental symptoms involves necessarily indirect psychopathological observation. Jaspers’ ambiguity is traced to his failure to distinguish clearly between direct and indirect psychopathological observation along with his excessive reliance on empathy. Relatedly, Jaspers’ ambiguity is also traced to his conflation of the semantics with the epistemology of psychopathological concepts representing patients’ “subjective” mental symptoms. These results apply also to contemporary phenomenological approaches to psychopathological diagnostic examination which maintain that patients’ abnormal mental experiences are invariably expressed in their overt behavior.

**Conclusions:**

Jaspers was right in stressing that psychopathological concepts of subjective mental symptoms represent patients’ genuine abnormal experiences irreducible to concepts representing their associated behavioral manifestations. Moreover, he was right in stressing the importance of the empathic ‘second person’ approach to patients’ mental experiences. However, he failed to recognize unambiguously that the epistemological access to patients’ mental symptoms, though enormously aided by empathy, remains mainly indirect and thus requires also a ‘third person’ approach to them. Overall then, clinical psychopathological examination requires both a ‘second’ and a ‘third’ person approach, as well as their judicious alternation during the diagnostic interview. Although focused on Jaspers’ essay, my critical analysis is also highly relevant to contemporary psychopathological approaches aiming to overcome the serious limitations of currently prevailing systems of diagnostic criteria of mental disorders.

## Introduction

Jaspers’ essay of 1912 “The phenomenological approach in psychopathology” is justly considered as a classic in clinical psychiatry [1,2]. Its lasting impact lies in Jaspers’ insistence that the accurate description of patients’ actual abnormal mental experiences involves a special method, namely the method of empathy (‘Einfühlung’).

The aim of this paper is to scrutinize critically Jaspers’ account of the epistemological role of empathy in psychopathological diagnostic investigation. This issue has been insufficiently clarified in a recent extensive and otherwise illuminating commentary on Jaspers’ essay ([3], pp 181–191). Owing to my exclusive focus on the epistemological status of empathy in clinical psychopathological diagnosis, I will leave completely aside here the issue of the undeniably crucial role of empathy in the context of psychotherapies. In particular, I will not address the issue of whether the adoption of a “phenomenological stance” can give rise to a distinctive kind of empathy (“radical empathy”) enabling the understanding of abnormal mental experiences in patients with severe mental disorders [4]. Phenomenological approaches stress the paramount importance of our embodied and contextualized interactions with others for understanding their mental states. Relatedly, they claim that most if not all mental experiences are expressed in behavior and thus are directly understood (e.g. [5]). The evaluation of these approaches lies well beyond the scope of the present work. However, I will deal briefly with the latter claim known as the ‘direct perception’ thesis of another’s mental states and argue that even if it were true in general, it would still not provide a fully adequate account of clinicians’ reasoning in the context of psychopathological diagnostic examination. The plan of the paper is the following: I first discuss Jaspers’ bipartition of mental symptoms in ‘objective’ and ‘subjective’. Then, I examine thoroughly Jaspers’ assertions on the exact epistemological role of empathy as the special method for the investigation of ‘subjective’ mental symptoms, and find them ambiguous. Furthermore, I elucidate the epistemological status of psychopathological examination as a prerequisite for the investigation of the exact role of empathy therein. I argue that psychopathological diagnostic examination involves mainly indirect scientific observation and that empathy, though important therein, has a mainly heuristic, not probative, value. Moreover, I propose an explanation of Jaspers’ ambiguity and a re-interpretation of his essay which restores its clarity. Finally, I explain why contemporary phenomenological approaches cannot provide a fully adequate account of the epistemological status of psychopathological diagnostic examination.

### Psychopathological diagnosis, mental symptoms and empathy

Jaspers partitions the set of mental symptoms into two major classes: the class of ‘objective’ and the class of ‘subjective’ symptoms. Moreover, he partitions further the ‘objective’ symptoms in three categories. The first subsumes those which are accessible to our unaided visual perception: patients’ spontaneous movements, appearance, observable actions etc. Furthermore, the second category subsumes those elicited by specific clinical tests, e.g. of intelligence or memory, and the third those which can be detected in the rational content of patients’ spontaneous verbal reports such as e. g. bizarre delusions. ‘Objective’ symptoms share in common that they can be detected through sensory perception and rational thought only, without any empathic involvement of the diagnostician. Several of Jaspers’ ‘objective symptoms’ are what nowadays are called clinical ‘signs’, i.e., clinical features of mental disorder directly observable or, if only indirectly observable, elicited through the application of appropriate standardized techniques without any recourse to patients’ subjective complaints and confidences. Those which can be identified by direct clinical observation can be described accurately with the aid of clinical observational concepts, e.g. psychomotor retardation, stupor, psychomotor excitement or psychomotor agitation. However, several of his other ‘objective symptoms’ require for their diagnostic detection indirect observation. For example, the administration of neuropsychological tests for the diagnostic detection of memory disturbances is an instance of indirect observation, since the accuracy of their findings depends essentially on the reliability and validity of these tests.

By contrast, “subjective” mental symptoms cannot be accessed in this manner, but only through empathy. Empathy, empathic understanding or *empathic actualization (‘Vergegenwärtigung*’) of patients’ mental experiences by clinical psychopathologists during the diagnostic interview is the proper method of investigation of subjective or properly mental symptoms. ‘Subjective symptoms cannot be perceived by the sense-organs but have to be grasped by transferring oneself, so to say, into the other individual’s psyche, that is, by empathy. They can only become an inner reality for the observer by his participating in the other person’s experiences (‘through co-*experience*/Mit*erleben’* in the original, Jaspers’ italics), not by any intellectual effort (Denken)’ ([1], p. 314, [2], p. 2).

The method of empathy aims at the understanding of patients’ mental experiences. Jaspers distinguishes between the *static* and the *genetic* understanding of patients’ mental experiences. Static understanding consists in the empathy-driven re-experience of patients’ actual mental experiences during the diagnostic interview, without any human prejudices or pre-conceived theoretical assumptions. As such, static understanding amounts to the accurate observation, description and diagnostic identification of abnormal mental experiences. By contrast, genetic understanding consists in the empathy-driven detection of the meaningful connections between patients’ abnormal mental experiences in their temporal unfolding, including the psychological impact of adverse life-events or processes on their current mental state and behavior.

### Jaspers on the epistemological role of empathy in psychopathological diagnosis

At several places of his essay, Jaspers claimed that clinicians’ empathic access to the various types of patients’ mental experiences is a *direct* process. Jaspers conceived of this process as *analogous* to the process of direct observation in the natural sciences and its presumed maximal accuracy, likening it to the process of vision, however with an ‘inner eye’: ‘We can grasp (subjective symptoms such as emotions and inner processes) immediately from their physical concomitants; these we take thus to “express” the underlying emotion’. (…) ‘For the actualization to ourselves of all these phenomenologically ultimate characteristics, we have such expressions as “seeing”, “viewing” (…) and so on. These expressions always denote the kind of ultimate concept-fitting experience which plays the same role in psychology as sensory perception plays in the natural sciences. Just as sense-perceptions are evoked by the demonstration of an object, so this meaningful empathic actualization will be evoked in us by (…) our immediate grasp of expressive phenomena and our self-immersion in other people’s self-description’ ([1], p. 319, [2], p. 4). ‘Phenomenology (…) views psychic events “as from within”, and brings them into immediate realization’ ([1], p. 326, [2], p. 8), ‘Only where something can be reduced to “reality” and becomes an immediate datum, i.e. becomes concrete, can it form the subject for phenomenology’ ([1], p. 323, [2], p. 7). Psychopathologists ‘acquire an unprejudiced direct grasp of these events as they really are’ ([1], p. 318, [2], p. 4). ‘Phenomenology deals only with the immediately given’ ([1], p. 323, [2], p. 6, slightly modified English translation). Moreover, in teaching psychopathology, the specialist ‘must make sure that’ (his students) ‘do not simply *think* along him, but they *see* along him in contact and conversation with patients and through their own observations. This “seeing” is not done with the senses, but through the understanding’ ([1], p. 318, [2], p. 4).

On the other hand, at other places of his essay, Jaspers stressed that empathy does not provide such an immediate access to patients’ mental experiences. Instead, clinicians should also have recourse to a variety of *indirect* methods such as ‘exploration by direct questioning of the patients and by means of accounts they themselves, under our guidance, give of their own experiences’ or patients’ ‘written self-descriptions’ ([1], p. 320, [2], p. 5). More precisely, ‘they are all those psychic experiences and phenomena which patients *describe* to us and which only become accessible to us at secondhand *(‘mittelbar’/*indirectly in the original with Jaspers’ italics) through the patient’s own *judgment* and presentation’ ([1], p. 314, [2], p. 2, Jaspers’ italics). Moreover, ‘subjective symptoms also include those mental processes which we have to infer (and ‘*interpret/deuten’* with italics in the original, omitted in the English translation), from fragments of the two previous kinds of data, manifested by patient’s actions and the way he (sic) conducts his life’ ([1], p. 314, [2], p. 2). ‘We have to be led, starting *from the outside* (italics in the original), to a real appreciation of a particular psychic phenomenon by looking at its genesis, the conditions of its appearance, its configurations, its context and possible concrete contents, also by making use of intuitive comparison and symbolization, by directing our observations in whatever ways may suggest themselves (…). The more numerous and specific these indirect hints become, the more well-defined and characteristic do the phenomena studied appear’ ([1], p. 318, [2], p. 4). Finally, even my quotation of Jaspers on the teaching of psychopathology is preceded by an analogy of psychopathological observation with *histological* observation, a standard example of *indirect* scientific observation through microscope. ‘A histologist will provide an exhaustive description of particular morphological elements, but he will do it in such a way as to make it easier for others to see these elements for themselves, and he has to presume, or else induce, this “seeing for oneself” in those who really want to understand him” ([1], p. 318, [2], p. 4).

In his opus magnum “General psychopathology” published the following year and regularly revised and expanded for almost 30 years, Jaspers dealt with the same issue in only two pages, referring the reader for a more detailed analysis to his essay of 1912 ([6], pp. 47–48). Therein, Jaspers stressed more clearly the mainly indirect mode of access to patients’ abnormal mental experiences through a whole battery of ‘external features’ ([6], p. 47). However, he persisted in claiming that psychopathologists should strive for ‘the unprejudiced *direct* grasp of the mental as it is’ ([6], p. 48, emphasis added).

Overall then, the elucidation by Jaspers of the epistemological role of empathy or static understanding in clinical psychopathological examination and diagnosis is far from clear. Granted that psychopathological concepts of mental symptoms represent real kinds of patients’ abnormal mental experiences and not their possible behavioral manifestations, it remains unclear whether empathy provides a direct or indirect mode of access to them. Of note, as a *psychological* process, this access might obtain *quasi-automatically* or rapidly and as such remain non-conscious, especially after long clinical training and experience. To the extent that Jaspers admits the necessity of psychopathologists’ recourse to these “numerous indirect hints”, he admits that empathic access is indirect, which however is in tension with his numerous claims to the contrary quoted above.

Thus, the complex epistemological operation of psychopathological diagnostic examination deserves further scrutiny in order to better appreciate the place and role of empathy within it. Accordingly, in the following section, I provide a brief characterization of psychopathological diagnostic examination, following the standard in contemporary philosophy of science distinction between direct and indirect scientific or, for that matter, clinical diagnostic observation [7]. Direct scientific observation bears on features accessible to unaided perception. By contrast, indirect scientific observation bears on features which are not so accessible, but are inferred with the help of hypotheses linking them to features accessible to unaided perception. The latter are then called indicators of the former and the hypotheses in question indicator-hypotheses. Although direct observation is usually less effortful than indirect observation, this is far from invariant: expert feature detection through indirect observation can become after long training almost “automatic” or effortless, whereas direct observation can at times require great effort.

### On the epistemological status of psychopathological diagnostic examination

The overwhelming majority of mental symptoms cannot be directly observed and “actualized” in clinicians’ consciousness, even if their presence may be evoked or suggested by the direct observation of patients’ appearance and behavior. This is as it should be, since all proper mental functions such as thought, emotion, memory, attention, perception, volition and motivation, along with their psychopathological disturbances, are not directly observable. Typically, mental symptoms are inferred during the diagnostic interview from patients’ spontaneous and elicited verbal descriptions of their mental experiences. Even spontaneously expressed mental symptoms in patients’ verbal self-descriptions need to be scrutinized through elucidatory questions before being diagnosed as such. This is so since a whole battery of generic conditions needs to be satisfied in order to ensure the *reliability* of patients’ self-descriptions, that is, their accurate reflection of patients’ underlying abnormal mental experiences. The most conspicuous of these conditions are the following: First, the patient is willing to cooperate and verbally reports sincerely her mental experiences *(C1*). Second, she is able to understand clinicians’ eliciting questions to this effect (*C2*). Third, she is able to identify, recall and report verbally her mental experiences with reasonable accuracy. This point has been raised by Langenbach as a limitation of Jaspers’ ‘phenomenology’ owing to the “dependency on patients’ abilities to describe exactly and to communicate their mental experiences” ([8], p. 217). Conversely, clinicians must disambiguate patients’ verbal reports on both their present and past mental experiences *(C3*). Fourth, the conditions under which the clinical interview takes place should help the patient to overcome her psychological inhibitions or reticence to talk about intimate and confidential personal matters *(C4*). Fifth, her answers are not obtained through suggestion by clinicians (*C5*).

Moreover, the very wording of the specific questions asked during the diagnostic interview is often crucial for the detection of mental symptoms. For example, it has been found that almost one third of patients with verbal auditory hallucinations expect bystanders to hear them also. Accordingly, the question pertaining to their diagnosis in the Structured Clinical Interview for DSM-IV [9] ‘do you hear things that other people do not hear?’ is clearly misleading, and needs re-phrasing as ‘do you hear things that other people around do not seem to hear, although perhaps you expect them to hear or even believe that they do?’ [10]. Last but not least, psychopathological diagnostic examination presupposes the mastering of a host of generalizations of two types: First, *definitions* of the concepts of mental symptoms ‘*patient x experiences symptom S, if and only if, x experiences jointly features F1, F2…,Fn’,* and, second, *diagnostic hypotheses* linking the indicators manifest in patients’ verbal reports to their underlying mental symptoms ‘*if patient x reports verbal material indicative of experiential features F1, F2,…,Fn, jointly characteristic of mental symptom S, and conditions C1-C5 obtain, then x has mental symptom S’.* Generalizations of the second type sound as crypto-tautologies but they are not, since the meanings of the concepts ‘reporting’ and ‘experiencing’ are clearly different: the former represents a *behavioral* process or activity whereas the latter a *mental* one. Moreover, only to the extent that conditions C1-C5 obtain are diagnostic inferences of this type logically valid.

Overall then, the pattern of psychopathological diagnostic examination in routine clinical practice is the following: From patients’ verbal reports of some features of their mental experiences and their empathic actualization in our consciousness, along with the mastering of basic psychopathological concepts and the reasonable satisfaction of conditions C1-C5, we *infer tentatively* that they are features of some known kind of mental symptom. For example, if the patient reports recurrent and persistent thoughts, impulses or images and conditions C1-C5 obtain, we conjecture that the latter are experiential features of obsessions. Next, we *deduce* that if they are features of that kind of mental symptom, they should also possess the remaining core features of that kind of symptom. In our example, we deduce that they should be invariably experienced as intrusive, inappropriate, anxiety-generating and impossible to ignore or suppress, despite being recognized as generated by one’s own mind. Then we investigate further these mental experiential features as well as their consistency and stability in search of additional relevant evidence in order to *test* our psychopathological diagnostic hypothesis. Furthermore, the elicitation of patients’ mental symptoms, along with the mastering of criteria or guidelines for the diagnosis of mental disorders, gives rise to alternative diagnostic hypotheses which are in turn tested following the same methodological pattern.

### Psychopathological diagnostic examination and the exact place of empathy

To be sure, patients’ ‘expressive phenomena’, i.e. their prevalent ongoing emotional experiences in the course of the clinical interview, can indeed be immediately ‘grasped from their physical concomitants’, such as facial expressions, tone of voice, eye-contact, spontaneous movements etc., by direct observation. However, emotions in general and their mental experience as feelings *cannot* be reduced to their associated observable behavioral, especially facial, manifestations which are only their clinical *indicators.* Indeed, emotions are global bodily changes and feelings their subjective experience in one’s consciousness [11]. Moreover, even this type of empathy, does not guarantee invariably the accuracy of our spontaneous ‘empathic actualizations’ and the resulting diagnostic judgments, as attested e.g. by clinical cases of persuasive simulators. This holds a fortiori for our investigation of patients’ abnormal mental experiences, possibly without any manifest ‘concomitants’, such as self-disturbances, beliefs, intentions, memories, impulses, or perceptions. This is indeed the case for most properly mental symptoms, e.g. passivity experiences, phobias, obsessions, delusions or hallucinations, as well as all for the totality of patients’ *past* abnormal mental experiences including their emotional experiences, all the more so since memory biases are pervasive. Here, we must indeed have recourse to Jaspers’ numerous ‘indirect hints’, along with a perfect mastering of psychopathological concepts, in order to form diagnostic hypotheses and then check them for accuracy against new clinical material. Moreover, the epistemological access this second type of diagnostic reasoning gives us to patients’ mental experiences is far from direct: assessing meticulously the reliability of patients’ verbal reports (conditions C1-C5 above) and identifying accurately the symptomatic constellations of their underlying experiential features is typically an inferentially highly complicated indirect process. Likewise, our diagnostic judgments are fallible and need to be further checked for accuracy.

What might be accessed directly are not patients’ mental symptoms as experienced by them, but *as tentatively re-experienced* by us. Conflating our mental *representations* of patients’ experiences with their *actual* experiences amounts to the conflation of our representations with the items represented. To be sure, Jaspers did not commit this fallacy, but several passages of his essay quoted above might be misinterpreted along these lines. Of note, the thesis of a direct and transparent epistemological access to one’s own mental states has been seriously challenged by psychological research (see e.g. [12]). Even granting the validity of this thesis, it remains still that clinicians’ immediately accessed self-representations of their patients’ experiences are always the products of an *indirect* and fallible process of imaginative *reconstruction* of patients’ unobservable experiences from their verbal and non-verbal behavior, along with the mastering of clinical psychopathological concepts. The experience of this process by expert clinicians as quasi-spontaneous implies neither its epistemological directness nor the accuracy of the resulting diagnostic judgments. Besides, in diagnostically complicated or atypical cases, even expert clinicians experience this process as reflective, laborious and inferentially indirect. The same holds also for the clinical investigation of novel abnormal kinds of mental experiences for which no descriptive psychopathological concepts are as yet available.

What distinguishes psychopathological clinical examination from other types of indirect scientific observation is the *additional* aid provided by our familiarity with other peoples’ mental life from our own *personal* mental life and our past *interpersonal* experiences, as well as our capacity to form vivid and comprehensive representations of the various kinds of mental symptoms. It is here I think that phenomenological approaches stressing the importance of our interactions with others in specific contexts for their empathic understanding are indeed clearly indispensable though insufficient as I will point out in the next section, at least in the context of psychopathological diagnostic examination. Moreover, the arts and the humanities, especially literature, can help refine our ability to imaginatively represent other peoples’ mental experiences from their own perspectives, all the more so since the range of our personal mental experiences is necessarily limited. Prominent phenomenological thinker Alfred Schutz has stressed that our understanding of the inter-subjective social world is mediated by our pre-reflective categorization of other people’s actions, motives, relations, or social roles, etc., in specific social life-situations (called by him, following Husserl, “typifications”, see e.g. [13]). More importantly, Schwartz and Wiggins have also stressed the ubiquity of typifications in the context of psychopathological diagnosis, acquired and refined by long clinical training and extensive personal experience of working with patients. It is through this training and experience that we acquire and refine these typifications involved in the diagnostic identification of the various kinds of mental symptoms and mental disorders. However, they have rightly warned that, contrary to the unreflective or “naïve” typifications in ordinary life, our psychopathological diagnostic typifications should be always subjected to critical scrutiny and tested for accuracy by clinical evidence [14]. Thus, all these additional aids *facilitate* our efforts to represent to ourselves patients’ mental experiences, investigate their connections to patients’ personality and life-conditions and assess the severity of their impact on patients’ personal, inter-personal and social life, however without rendering our access to them direct. More accurate representations of patients’ abnormal mental experiences might also require the formation of more refined clinical psychopathological concepts than those figuring in the diagnostic criteria of DSM [15]) (see e.g. [16,17]). This might well be the case in the field of depressive disorders whereby the delineation of distinct varieties of depressed mood now conflated in the DSM would facilitate both their clinical and biological investigation and help improve their diagnostic validity. Incidentally, the necessary investigation of the connections between patients’ presumably abnormal mental experiences and their overall life-conditions and personality calls into question Jaspers’ sharp distinction between static and genetic understanding. Furthermore, empathy, though eminently helpful in the framing of diagnostic hypotheses is clearly insufficient for their validation during the clinical interview. In other words, empathy has a mainly *heuristic*, not a *probative* value. However, this undeniable heuristic advantage comes at a price: we tend to project upon others our own social stereotypes and prejudices, running thus the risk to form biased or distorted ‘actualizations’ of their mental experiences. Although Jaspers did not claim that empathy has a probative value, again his ambiguity on its epistemological role might be misinterpreted along these lines, all the more so since he also claimed that the accessibility of patients’ mental experiences to clinicians’ empathic static understanding warrants their categorization in three classes: fully understandable (strongly similar to our own mental experiences), only partly understandable (similar in kind though markedly dissimilar in intensity or clarity to our own mental experiences) and, finally, completely un-understandable or qualitatively dissimilar to normal mental experiences (e.g. experiences of passivity and external control in schizophrenia) ([1], p. 321, [2], p. 5). Relatedly, German Berrios has criticized Jaspers for his “exaggerated reliance on the powers of the observer and on the use of his/her cognitive and social context to decide on whether or not a given mental state is ‘comprehensible’” ([18], p. 319). Besides, the serious shortcomings of our intuitive understanding of our fellow humans are now well attested (see e.g. [19]). So far, empirical research shows that humans display, in their ordinary encounters with other people, rather low levels of empathic understanding, ranging from 25% to 35% [20].

### A proposed “diagnosis” and “treatment” of Jaspers’ ambiguity

I submit that the logical contrariety or, at least, the ambiguity in Jaspers’ essay stems from two main sources. The first is his lack of any clear distinction between direct and indirect observation. This distinction would hold even if it were construed as a mere difference of degree along a continuum, not of kind. Indeed, proper mental experiences, by their very nature, would be placed far closer to the pole of the indirectly observables than to the pole of the directly observables. Despite his nominal use of the terms ‘direct’ and ‘indirect’, I presume that Jaspers thought that all observation is eventually direct observation. This is strongly suggested by his misleading analogy between our sense-perception of the outer physical world and our empathic understanding of the inner mental world. Accordingly, Jaspers was tempted to construe psychopathological examination of mental symptoms as a novel kind of direct observation, however ‘with an inner eye’. Jaspers’ misunderstanding of histological observation as an example of direct (visual) observation reinforces my presumption. However, his motivation was perfectly sound: mental experiences and symptoms are not identical to their possible overt manifestations in patients’ behavior. Accordingly, the psychopathological concepts intended to represent these mental experiences and symptoms differ from the concepts representing their possible overt manifestations. This is a difference in their meaning, i.e. their sense and reference. Their reference is what they intend to represent (their referent) and their sense a description of its main features. However, this difference is independent from the mode of our epistemological access to their referents. In particular, the (direct) reference of the concepts of properly mental symptoms (‘subjective’ symptoms in the terminology of Jaspers) to patients’ inner experiences does not entail that our access to them is inferentially direct. For example, the concept of obsession represents directly a recurrent type of patients’ mental experiences with particular experiential features (see above), not their possible overt behavioral manifestations. However, this does not render our diagnostic access to patients’ obsessions inferentially direct. In other words, this is a difference in the *semantics, not* the *epistemology* of our psychopathological concepts and hypotheses formulated with their help. Here lies the second source of Jaspers’ ambiguity on the epistemological status of empathy: Jaspers conflated the *semantic* category of what basic psychopathological concepts represent, namely patients’ unobservable kinds of mental symptoms (their reference) with the *epistemological* category of our *mode of access* to their referents. At times also, he seems to conflate these semantic and epistemological categories with the *psychological* category of clinicians’ subjective certainty ([1], p. 315, [2], p. 2).

Although focused on Jaspers’ classic essay, my analysis of the epistemological status of psychopathological diagnostic examination and the role of empathy therein exceeds by far the exegesis of Jaspers’ classic essay and is highly relevant to contemporary psychopathological approaches aiming to overcome the serious limitations of currently prevailing systems of diagnostic criteria of mental disorders. More precisely, one such innovative post-Jaspersian phenomenological approach in psychopathology contests the very distinction between patients’ inner abnormal mental experiences and their outer manifestations, stressing the *expressive* nature of *all* mental symptoms (see e.g. [21]). This approach stems from a rich philosophical phenomenological tradition including the work of Edmund Husserl, Martin Heiddegger, Maurice Merleau-Ponty, Max Scheler, Edith Stein, and Alfred Schutz, further developed and defended by contemporary philosophers, notably by Shaun Gallagher and Dan Zahavi (see e.g. [5]). According to this approach, mental states are directly expressed in one’s expressive behavior and thus, our understanding of others is inferentially direct. This approach to social cognition rejects the ‘third-person’ observational stance towards others and favors instead a ‘second person’ interactive stance whereby mental states are expressed in embodied and contextualized behaviors. I cannot deal here with this important approach to social cognition and inter-subjective understanding which challenges both mainstream approaches to our ability to ascribe and understand the mental states of our fellow humans (“theory of mind”), namely the “theory-theory” and the “simulation theory” (see e.g. [22]). However, all these theories bear on the development and the underpinnings of our generic human capacity to understand others. By contrast, as I tried to show, psychopathological diagnostic examination in the context of the clinical interview is not merely a social human interaction but, in addition, a specialist endeavor with the aid of concepts afforded by general and clinical psychopathology and a specific aim, namely a maximally accurate and comprehensive psychopathological diagnosis. Moreover, this endeavor requires the mastering of psychopathological concepts, the thorough investigation of patients’ abnormal mental experiences with the aid of indicator-hypotheses linking these experiences to their verbal and non-verbal manifestations, as well as the satisfaction of a whole set of further conditions (see conditions C1-C5 above). As such, it involves not only the generic ‘second person’ stance of a simple interactive social encounter, but also a ‘third person’ observational stance. It goes without saying that it requires also clinicians’ ability to adopt both stances and switch flexibly from the one to the other during the diagnostic interview. It is in this specific context that I find problematic one of the main tenets of the phenomenological approach, namely the thesis of an invariant relation of expressivity between one’s mental experiences and behavioral manifestations since it implies that patients’ psychological states and experiences are directly observable and understood. As a result, this approach sees no need for any distinction between direct and indirect psychopathological observation and thus incurs the risk of construing again all psychopathological observation as direct observation “with an inner eye”. Moreover, since this approach admits the reality of patients’ mental experiences, it incurs also the risk of conflating the semantics with the epistemology of diagnostic psychopathological concepts and hypotheses. If, as I tried to show at some length, most proper mental symptoms lack distinctive behavioral manifestations, this approach cannot be fully adequate, at least in the field of psychopathological diagnostic examination. Besides, the ‘direct perception’ thesis about our mode of understanding others has also been challenged in the philosophy of mind. In particular, it has been pointed out that it cannot account for the under-determination of mental states by behavioral evidence: the same behavioral evidence is compatible with different underlying mental states. Thus, behavioral evidence is insufficient to ascertain and identify others’ beliefs and intentions (see e.g. [23,24]. Overall then, the phenomenological ‘second person’ approach, though a necessary ingredient of the psychopathological diagnostic examination is clearly insufficient and needs to be supplemented by a ‘third person’ approach along the lines of my epistemological analysis.

## Conclusions

I have examined Jaspers’ account of the epistemological role of empathy in the context of the clinical diagnostic examination pointing out the ambiguity of his main claims. Then, I have tried to elucidate the epistemological status of psychopathological diagnostic examination with the aid of the standard in contemporary philosophy of science distinction between direct and indirect observation. This elucidation was a prerequisite for the investigation of the exact epistemological role of empathy within the context of psychopathological diagnostic examination. My main result was that clinical diagnostic examination of properly mental symptoms (Jaspers’ “subjective” symptoms) is mainly indirect psychopathological observation. I have then traced Jaspers’ ambiguity to his failure to distinguish clearly between direct and indirect psychopathological observation as well as between the semantics and the epistemology of clinical psychopathological concepts. Disentangling these issues helps remove Jaspers’ ambiguity and restore the coherence of his account. Thus, Jaspers was right in claiming that psychopathological concepts of mental symptoms represent directly real abnormal experiences and thus, are semantically irreducible to concepts representing their actual or possible behavioral manifestations. However, he failed to recognize clearly that our epistemological access to these experiences in the context of psychopathological diagnostic examination is mainly *indirect.* My analysis of the epistemological status of psychopathological diagnostic examination and the role of empathy therein is also highly relevant to recent innovative phenomenological approaches aiming to overcome the serious limitations of currently prevailing systems of criteria for the diagnosis of mental disorders.

## Competing interest

The author declares that he has no competing interest.

## Authors’ information

Panagiotis Oulis, M.D., Ph.D., MPH, Associate Professor of Psychiatry.
